# Antioxidant Supplementation in the Treatment of Neurotoxicity Induced by Platinum-Based Chemotherapeutics—A Review

**DOI:** 10.3390/ijms21207753

**Published:** 2020-10-20

**Authors:** Jelena S. Katanic Stankovic, Dragica Selakovic, Vladimir Mihailovic, Gvozden Rosic

**Affiliations:** 1 Institute for Information Technologies Kragujevac, Department of Science, University of Kragujevac, Jovana Cvijica bb, 34000 Kragujevac, Serbia; jkatanic@kg.ac.rs; 2 Faculty of Medical Sciences, Department of Physiology, University of Kragujevac, Svetozara Markovica 69, 34000 Kragujevac, Serbia; dragica984@gmail.com; 3 Faculty of Science, Department of Chemistry, University of Kragujevac, Radoja Domanovica 12, 34000 Kragujevac, Serbia

**Keywords:** platinum-based drugs, cisplatin, carboplatin, oxaliplatin, neurotoxicity, peripheral neuropathy, antioxidants

## Abstract

Cancer represents one of the most pernicious public health problems with a high mortality rate among patients worldwide. Chemotherapy is one of the major therapeutic approaches for the treatment of various malignancies. Platinum-based drugs (cisplatin, oxaliplatin, carboplatin, etc.) are highly effective chemotherapeutic drugs used for the treatment of several types of malignancies, but their application and dosage are limited by their toxic effects on various systems, including neurotoxicity. Simultaneously, researchers have tried to improve the survival rate and quality of life of cancer patients and decrease the toxicity of platinum-containing drugs by combining them with non-chemotherapy-based drugs, dietary supplements and/or antioxidants. Additionally, recent studies have shown that the root cause for the many side effects of platinum chemotherapeutics involves the production of reactive oxygen species (ROS) in naive cells. Therefore, suppression of ROS generation and their inactivation with antioxidants represents an appropriate approach for platinum drug-induced toxicities. The aim of this paper is to present an updated review of the protective effects of different antioxidant agents (vitamins, dietary antioxidants and supplements, medicaments, medicinal plants and their bioactive compounds) against the neurotoxicity induced by platinum-based chemotherapeutics. This review highlights the high potential of plant antioxidants as adjuvant strategies in chemotherapy with platinum drugs.

## 1. Introduction

A “honeymoon” with platinum-based compounds in chemotherapy that officially started by pronouncing cisplatin as “the drug of the century” more than 50 years ago has gradually been overshadowed by very serious therapeutic limitations of those drugs. Besides the inherited resistance to treatment with any of the currently approved platinum agents, each of them has a number of clinically confirmed side-effects, ranging from minor to dose-limiting. The fact that almost half of all the patients who receive anticancer chemotherapy are treated with a platinum drug [1] gives a good insight into broad-spectrum platinum-based chemotherapeutics adverse effects.

## 2. Therapeutic Indications for Platinum-Based Chemotherapy

For decades various platinum-based compounds were employed as an important part of the combination chemotherapy regimens used to treat different types of solid tumors. However, there are only three platinum-based medications that are used throughout the world for the cancer treatment: cisplatin (*cis*-diamminedichloridoplatinum II), carboplatin (*cis*-diammine-1,1-cyclobutanedicarboxylateplatinum), and oxaliplatin (*trans*-R,R-cyclohexane-1,2-diamineoxalato- platinum II), while some other platinum-based therapeutics are approved only in individual countries, such as heptaplatin (Korea), lobaplatin (China), miriplatin (Japan), and nedaplatin (Japan) [2].

Since 1979 cisplatin has been widely used (along with other antineoplastic drugs) in the treatment of various malignancies: lung [3], ovarian [4], testicular [5], breast [6] and brain cancer [7], sarcomas [8], and lymphomas [9]. Starting in 1989, carboplatin confirmed clinical relevance as an antineoplastic agent (in combination with other chemotherapeutics) for advanced ovarian carcinoma [10], head and neck cancer [11], and lung cancer [12]. The latest worldwide accepted platinum-based chemotherapeutic (2002), oxaliplatin, is used as a part of the therapeutic protocols for metastatic colorectal cancer [13], advanced gastric [14] and ovarian cancer [15].

## 3. Side Effects of Platinum-Based Compounds in Clinical Practice

Like for the other chemotherapeutic drugs, the basic cytotoxic effect of platinum-based compounds (DNA damage) is not restricted only to target tissue (tumor cells), but is also affecting numerous organ systems in patients receiving chemotherapy, resulting in a variety of side effects. Based on a similar pathophysiological background, the adverse effects of platinum-based chemotherapeutics may be classified in certain categories of toxicities according to their clinical manifestations. The most commonly described types of side effects associated with platinum-based treatment are usually classified as nephrotoxicity, hepatotoxicity, neuro- and ototoxicity, cardiotoxicity, hematological toxicity, and gastrointestinal toxicity. However, patients’ responses to chemotherapy toxicity, including adverse effects of platinum-based compounds, are significantly determined by several factors, such as age, gender, drug administration schedule and performance status [16].

Nausea and vomiting are considered as the most common clinical manifestations of side effects following cisplatin administration. Strongly depending on the applied dose, this effect of cisplatin, which can be successfully abolished by antiemetic action of 5-HT3 antagonists [16], is found more often than in chemotherapeutic protocols with oxaliplatin and carboplatin [17].

Nephrotoxicity represents the main limitation for chemotherapeutic protocols that involved platinum-based compounds which is not surprising due to the fact that kidneys are the main route for platinum compounds excretion. Although the platinum-based drugs affect all three key kidney functions (filtration, reabsorption, and excretion), the two most common nephrotoxic side effects of cisplatin are acute kidney injury (also known as acute renal failure) and hypomagnesemia, which is reported to affect up to 90% of cisplatin-treated patients [2]. However, the comparison of toxicities for three platinum-containing chemotherapy regimens confirms the lower nephrotoxicity manifestations of both carbo- and oxaliplatin when compared to cisplatin [18].

Hepatotoxicity is also considered as one of the most frequent adverse effects of platinum-based compounds administration in clinical practice. Morphological alterations accompanying platinum-based compounds application are manifested as necrosis, degeneration of hepatocytes, and increased inflammatory response [19], as well as a consequent increase in hepatic enzymes and bilirubin [20]. Long-term survival analysis of platinum-based compounds-induced hepatotoxicity showed that liver damage was more pronounced following cisplatin administration when compared to carboplatin [21], while drug-induced hepatotoxicity manifestations accompanying oxaliplatin therapy were predominantly restricted to hepatic vascular injury [22].

**Figure 1:**
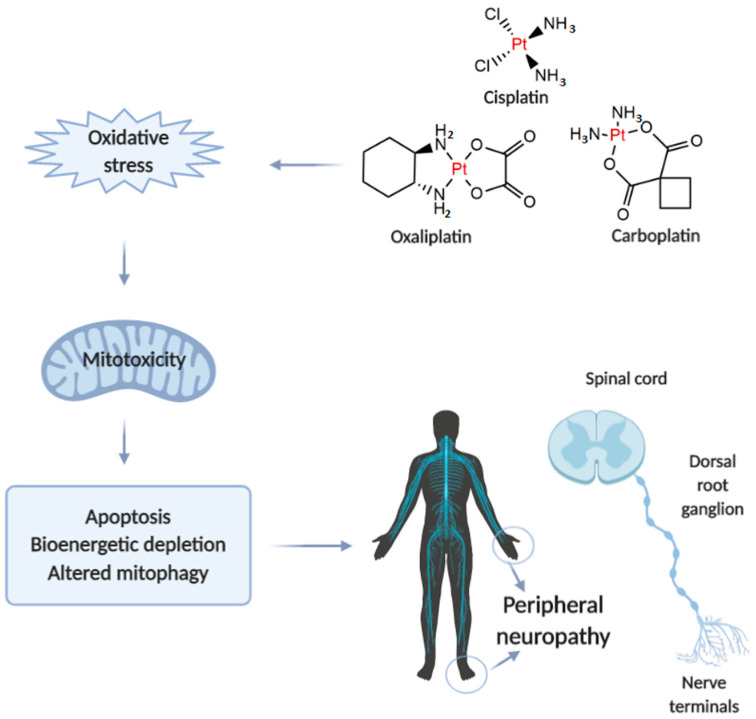
Illustrative presentation of the development of platinum-based drug-induced peripheral neuropathy. Created in BioRender.com.

Neurotoxicity in response to platinum-based therapy is the leading clinical entity, aside from nephro- and hepatotoxicity that usually hampers platinum-based chemotherapy (Figure 1). The most frequently reported manifestations of neurotoxicity are due to the clinical appearance of peripheral neurotoxicity (numbness, tingling, or paresthesia in fingers and/or toes). With prolonged treatment, they gradually lead to disturbance of proprioception, which may result in ataxic gait [23]. The clinical manifestations of encephalopathy accompanying platinum-based therapy usually appear with an increase in cumulative dose [24]. Sensory manifestations of platinum compounds-induced neuropathy are often accompanied by ototoxicity [25].

When comparing to the individual extension of neurotoxic manifestations for platinum-based drugs, it has been shown that carboplatin neurotoxicity is negligible compared to cisplatin and oxaliplatin. However, carboplatin, particularly when applied in high doses, can lead to the development of neurotoxic manifestations that may become irreversible in 30-50% of patients.

The evaluation of mechanisms underlying platinum-induced peripheral neurotoxicity (PIPN), in both in vivo and in vitro studies, confirmed that those compounds after passing the neuronal membrane initiate several proapoptotic phenomena including the activation of p53, Bax translocation, cytochrome c release, as well as the activation of caspase-3 and 9 [26]. Similar to the beneficial action of platinum compounds on tumor’s nuclear DNA, the undesirable impact has been observed on naïve cells mitochondrial DNA which results in inhibition of replication and translation, with consequent respiratory chain disturbance and energy deficiency [27]. Finally, the platinum compounds-induced mitochondrial dysfunction resulted in increased reactive oxygen species (ROS) production (with oxidative damage) and intracellular calcium accumulation [27]. Furthermore, the observed intracellular calcium accumulation was potentiated via up-regulation of calcium N channels induced by platinum compounds, that additionally potentiated apoptotic mechanisms [28]. An additional transmembrane mechanism is involved in the neurotoxic effect of platinum compounds on peripheral nerves. Namely, it has been reported that cisplatin-induced neurotoxicity increased expression of TRPA1 receptors in dorsal root ganglia that resulted in hyperalgesic response to thermal stimuli [29].

Interestingly, although it is still very questionable how cisplatin passes through an intact blood-brain barrier, there are certain literature data considering the central manifestations of neurotoxicity induced by platinum compounds. It has been shown that cisplatin administration strongly affects CNS progenitor cells and oligodendrocytes inducing the apoptotic events in hippocampal dentate gyrus and corpus callosum [30]. Only a few studies implicate that several mechanisms of platinum compounds action in CNS, including oxidative damage, inflammation and apoptosis, may be the cause of behavioral alterations manifested as increased anxiety [31], depression [32], as well as cognitive dysfunction [33].

Since there is a plethora of evidence that oxidative damage is crucially involved in the mechanisms of platinum-based compounds toxicities, it is not surprising that an enormous effort has been put in order to increase the safety of platinum-based therapy regimens by promoting the antioxidant supplementation as potentially protective interventions during the chemotherapy protocols-based platinum-containing antitumor agents.

## 4. Classification of Antioxidants

The root cause of many side effects and organ injury of platinum-based drugs is the generation of reactive oxygen species (ROS) [2]. The increase of ROS production associated with mitochondrial dysfunction in neurons was described as a potential mechanism of platinum drug neurotoxicity. The damage of mitochondrial DNA and interrupt RNA expression of Cytochrome B induced by platinum drugs applications give rise to disruption of neuronal mitochondrial function and overproduction of reactive oxygen species [34]. Therefore, antioxidant supplementation for the reduction of ROS, or alleviation of the effects of ROS, could also have significant influence on the neurotoxicity side effects of platinum drugs.

Oxidation reactions are very common in our body and they have a role to change a chemical substance, but also could produce highly reactive free radicals [35]. Xenobiotics, including drugs, represent pro-oxidant compounds and risk factors for the overproduction of free radicals. This process may lead to damage to biological molecules in their reaction with free radicals which tend to capture electrons from other molecules to stabilize themselves. All these reactions disrupt the structure and function of many tissues and organs, as well as the equilibrium between free radicals and antioxidants in the body [36]. The human body possesses a sophisticated and complex antioxidant protection system consisting of a variety of endogenous and exogenous originated substances which act interactively and synergistically, whether to actively inhibit oxidation reactions or to inhibit oxidation indirectly [37].

There are different definitions of antioxidants; the most comprehensive definition describes antioxidants as substances that directly scavenge free radicals, inhibit their production, or indirectly act to up-regulate antioxidant defenses. Furthermore, antioxidant compounds should have the ability to form a new stable radical that is inactive for further oxidation, after scavenging the free radical [38,39]. Antioxidant protection mechanisms in humans include three lines of defense: preventive antioxidants, radical-scavenging antioxidants, and repair and *de novo* antioxidants. There is also an adaptation mechanism of antioxidant defense that is referred to as ‘the fourth line of defense’ [40,41].

The first line of defense includes preventing mechanisms for biological molecules damage by suppressing or preventing the formation of free radicals and their derivatives. This defense line involves enzymatic antioxidants (Table 1) that catalyze the disproportionation reaction of reactive species such as superoxide anion (superoxide dismutase; SOD) or hydrogen peroxide (catalase; CAT, glutathione peroxidase; GPx, or glutathione reductase; GR). Also, the first line of antioxidant defense consists of proteins presented in blood plasma such as albumin, transferrin, ferritin, myoglobin, ceruloplasmin, and metallothioneins representing non-enzymatic antioxidants which bind polyvalent metal ion which may involve in redox reaction and produce free radicals. The second defense line consists of non-enzymatic molecules (Table 1) represented by vitamins (vitamin A, C, E, and K), glutathione (GSH), coenzyme Q10 (CoQ10), uric acid, melatonin, bilirubin, and alpha-lipoic acid (ALA) that scavenge ROS or reactive nitrogen species (RNS). The group of antioxidants that repair or eliminate structural damage of biomolecules caused by free radicals is classified as the third line of antioxidant defense (de novo antioxidants). This group mainly includes DNA repair enzyme systems, lipase, and proteolytic enzymes which can repair damaged molecules [40,42,43]. Furthermore, there is another important adaptation mechanism may be regarded as the fourth defense line that is activated by reaction and production of free radicals inducing the formation and transport of appropriate antioxidant enzymes to the right site. This mechanism includes, e.g., the formation of catalase and superoxide dismutase induced by ROS overproduction [40,41,44].

The human antioxidant defense mechanisms include both non-enzymatic and enzymatic antioxidants groups classified according to the molecular structure which forms endogenous antioxidant systems. But the human endogenous antioxidant system does not suffice, and the production of non-enzymatic antioxidants can be hardly producing in the human body. Therefore, there are exogenous non-enzymatic antioxidants (Table 1) such as mineral elements, nutritional antioxidants, and antioxidants obtained from natural resources (phytochemicals/phytonutrients) [36,39]. Exogenous non-enzymatic antioxidants extensively used in medicine and industry are divided into natural and synthetic. In view of the latest suspicions that synthetic antioxidants exert a noxious effect on human health, more attention is paid to natural antioxidants found in fruits, vegetables, nuts, and cereals, as well as medicinal and dietary plats for the use in food, cosmetic, and as therapeutics. The research studies carried in the field of natural antioxidants showed that phenolic compounds are the most studied phytochemicals. Among them, phenolic acids and flavonoids represent the most popular group of phenolic compounds because they may have excellent antioxidant activities but also good antimicrobial, anticancer, anti-inflammatory, and hormonal activities [44,45]. In this review, the authors aimed to summarize the most prominent research articles in terms of platinum-induced neurotoxicity and its treatment with different antioxidant supplementation. Scopus and PubMed Central databases were searched for published studies relevant to this matter, from 2010 until 2020. Since some papers referred to valuable older references, several were included in this review.

## 5. Antioxidants in the Treatment of Platinum-Based Chemotherapeutics-Induced Neurotoxicity

### 5.1. Vitamins, Minerals, and Dietetic Supplements

According to the World Health Organization recommendations nutrition is one of the major modifiable determinants of chronic disease [46]. Some essential nutrients, micronutrients, minerals, and trace elements, as well as nutritional supplements, beside their primary role for adequate functioning of an organism, possess anti-inflammatory, antihyperalgesic, and antioxidant effects through which they can influence on a chronic disease. One of the preventive and therapeutic strategies for alleviating neurotoxicity side effects in patients receiving platinum-based chemotherapeutics is the use of supplementation including vitamins, minerals and trace elements, as well as dietary supplements with antioxidant activity (Table 2) [47,48,49].

**Figure 2:**
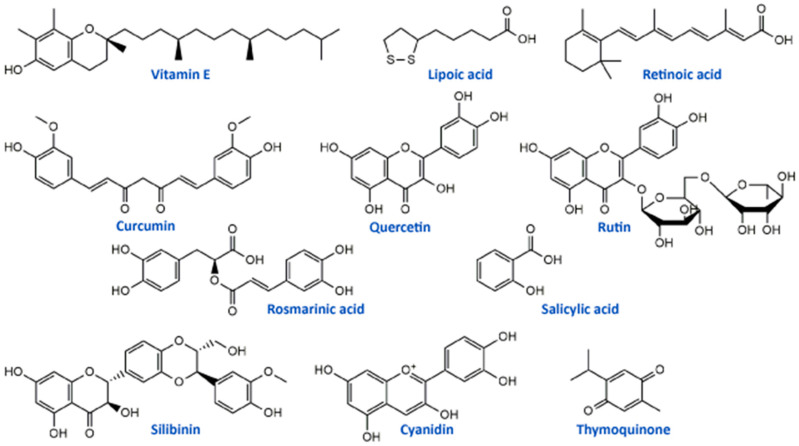
Chemical structures of some supplements and natural antioxidants (from Table 2 and Table 3) that exhibited neuroprotective potential against platinum drugs-induced neurotoxicities.

Vitamin E (Figure 2) is a potent antioxidant with high ability to neutralize ROS and protect cells and subcellular structures from lipid peroxidation [93]. Several studies have reported that cisplatin-induced neuropathy and vitamin E deficiency neuropathy manifest similar clinical and neuropathologic features [54,94]. Vitamin E is one of the most studied natural antioxidants in the prevention and therapy of the toxic effects of platinum drugs including fundamental and clinical studies. In vivo study on cisplatin-treated mice showed that vitamin E protected the peripheral nerve, decreased systemic toxicity, and increased antioxidant defense without interfering with its antitumor activity of cisplatin [52]. A randomized, placebo-controlled trial including 108 patients treated with cisplatin chemotherapy and vitamin E supplementation (400 mg/day) showed a significantly lower incidence of neurotoxicity in patients receiving vitamin E than in those receiving placebo. This phase III study provided evidence that vitamin E should be adopted in patients receiving cisplatin-based chemotherapy for reduction of risk of developing signs or symptoms of neurotoxicity [54]. Similar results were obtained in the study conducted by Argyriou et al. [53], suggesting the significantly lower incidence of neurotoxicity in patients with vitamin E supplementation (600 mg/day) during cisplatin chemotherapy. However, phase III trial study conducted in patients with neurotoxic chemotherapy (cisplatin, carboplatin, oxaliplatin, or a combination) supplemented with vitamin E (400 mg)/placebo showed no statistically significant difference between the incidence of sensory neuropathy in the studied groups (vitamin E and placebo) [55]. Also, studies designed to assess the influence of vitamin E supplementation (400 mg/day) for the prevention of oxaliplatin-induced peripheral neuropathy did not demonstrate any significant decrease in the incidence of peripheral neuropathy. Similar results were obtained in another study conducted by Salehi et al. [57]. The differences in numbers of patients included in clinical trials may be a possible explanation for the contrasting results.

Retinoic acid (Figure 2, a metabolite of vitamin A) has also been studied as an antioxidant supplement in cisplatin-induced neuropathy in an animal model and patients. Tredici et al. [50] in the study investigating the prevention of retinoic acid on cisplatin-induced neurotoxicity in rats reported that retinoic acid-induced only a mild generalized protective effect without significant influence on morphometric, electrophysiological, functional and analytical disturbance on dorsal root ganglion neurons caused by cisplatin application. On the other hand, in the study of Arrieta et al. [51] retinoic acid reduced cisplatin-induced neuropathy in rats, reduced the electrophysiologic alterations and nerve growth factor in serum in patients with non-small-cell lung cancer with standard treatment based on cisplatin and paclitaxel.

Thiamine pyrophosphate and water-soluble formulation of provitamin coenzyme Q10 (WS-CoQ10) has also been studied for neurotoxicity prevention. The neuroprotective effect of WS-CoQ10 was tested on PC12 cells exposed to cisplatin. These cells possess neuronal cell features and respond positively to nerve growth factor. WS-CoQ10 showed neuroprotective effects on the PC12 cells, protection from cisplatin-induced DNA damage, and an increase in the total intracellular GSH [58]. The application of thiamine pyrophosphate significantly improved oxidative parameters in the brain tissue of rats treated with cisplatin [59].

Selenium is a trace element that has an important role in cellular redox regulation and the protection of cellular components from oxidative damage. Selenium was shown to possess moderate neuroprotective effects on the cisplatin neurotoxic effect in rats. The study showed that selenium administration partly reverses nerve conduction velocity, the amplitude of compound action potential, and the number of axons in experiments with cisplatin application in rats [49].

Calcium and magnesium (Ca/Mg) infusions were suggested as supplementation in platinum-based drugs-induced peripheral neuropathy. Oxaliplatin is the most researched among them as chemotherapeutic agents because its metabolite oxalate has the potential to chelates both Ca and Mg ions disrupting the function of ion channels in nerve membranes [47]. The hypothesis that an increase of extracellular Ca and Mg may prevent or ameliorate oxaliplatin-induced neurotoxicity was approved in cell-based and animal studies [60,61]. There have been numerous clinical trials designed to evaluate the benefit of Ca/Mg infusions for decreasing oxaliplatin-induced neuropathy. Gamelin [95] concluded that Ca/Mg infusions reduced the incidence and intensity of acute oxaliplatin-induced symptoms in patients. Later studies did not confirm this observation, Loprinzi et al. [62] in the clinical study of intravenous Ca and Mg application for prevention of oxaliplatin-induced sensory neurotoxicity showed that Ca/Mg did not substantially decrease oxaliplatin-induced acute neuropathy.

Alpha lipoic acid (ALA) is one of the most studied dietary antioxidant supplements in the literature. This molecule (Figure 2) is essential for cell energy production in the Krebs cycle and represents endogenous antioxidant with free radical scavenging capability and the possibility to activate enzymes that reduce oxidative stress [65]. In an in vitro model using primary cultures of dorsal root ganglion, ALA protected these cells from cisplatin toxicity through its antioxidant and mitochondrial regulatory functions [63]. Also, another in vivo study on rats showed that ALA (100 mg/kg/day) restored conventional conduction velocity and conduction velocity distribution disturbed by cisplatin application [64]. Different clinical studies showed inconsistent results for use of ALA supplementation in cisplatin and oxaliplatin chemotherapy-induced peripheral neuropathy. Gedlicka et al. [66,67] in small clinical studies reported that ALA application in patients with established neuropathy secondary to the oxaliplatin/cisplatin treatment reduced the intensity of symptoms. On the other hand, Guo et al. [65] concluded in a randomized, double-blind, placebo-controlled trial with 243 patients that ALA was ineffective in preventing neurotoxicity caused by oxaliplatin or cisplatin. All these studies suggest that ALA may have benefits in platinum drug-induced neuropathy, but there is a need for deeper investigation, especially regarding clinical studies.

Melatonin is a pineal hormone, also known as a free radical scavenger, which manifested protection activity against oxaliplatin-induced alterations in motor activity and muscular strength in rats. Besides, this supplementation inactivated Bcl-2 and caspase 3 apoptotic protein, as well as decreased Cytochrome c release and modulated oxidative stress in rats’ brain caused by oxaliplatin application [71]. Tuncer et al. [64] reported a lower influence of melatonin on the modulation of cisplatin-induced neurotoxicity in rats compared to ALA.

Yehia et al. [68] reported that carnosine, an endogenous dipeptide occurring naturally in humans, reduced the symptoms when provided as a supplement to cancer patients with oxaliplatin-induced peripheral neuropathy by decreasing NF-κB and TNF-α, oxidative stress (reduced MDA and increased Nrf-2) and apoptosis (reduced caspase-3 activity).

D-Methionine is also one of the effective antioxidant supplements against cisplatin-induced neurotoxicity in experimental animals. It showed a positive influence on the cortical neurons damage in an in vitro model of cortical networks [72], prevented the decreased neurogenesis in the hippocampus of the adult rats [73], regulated electrophysiological recordings, and increased hippocampal neurogenesis in rodents after cisplatin application [74]. According to Gopal et al. [72], the optical isomer L-methionine was less protective on cisplatin-induced damage in cortical networks from mouse embryos compared to D-methionine. Also, some evidence showed that taurine (free intracellular amino acid also present in some food) has protective effects on cisplatin-induced toxicity modulating inflammation and oxidative damage. An in vivo study conducted on rats with cisplatin treatment confirmed significant amelioration of behavioral performance and antioxidant parameters in brain tissue, as well as a decrease in acetylcholinesterase activity in taurine-treated groups [75]. These amino acids with very promising results in fundamental studies have the potential for further investigations as nutritional supplements in clinical trials as co-adjuvant therapies in the reduction of neuropathy symptoms in patients with platinum-based drugs chemotherapy.

There are some studies about the use of different antioxidants which are usually not present or which are present in small amounts in the human diet, against cisplatin-induced neurotoxicity. Song et al. [69] reported that ergothioneine improved the learning and memory dysfunctions in mice treated with cisplatin, followed by the improvement of antioxidant status and the decrease of acetylcholinesterase activity in brain tissue. Ethoxyquin, a compound used as an antioxidant in animal feed, also showed protective effects against cisplatin-induced neurotoxicity in in vitro and in vivo studies [70]. Further safety and clinical studies are needed if this type of antioxidants is to be used as potential nutritional supplements adjuvant to neurotoxic chemotherapy.

### 5.2. Clinically Used Medications

The injurious effects of platinum-based drugs, especially a sensory peripheral neuropathy, are primarily dose-limiting, but they also can affect the quality of life of the patients. There are many possible therapies for the treatment of PIPN although none of them is completely efficient. That is the main reason for numerous investigations, presented in Table 2, aiming to develop promising adjuvant therapy for PIPN, without interfering with platinum cytostatic activity [96,97].

Amifostine is an organic thiophosphate that has cytoprotective and detoxicant activities. It is generally an inactive prodrug which activates after dephosphorilation in plasma membrane with alkaline phosphatase. When active metabolite enters the cell it act like free radical scavenger protecting DNA and cellular membranes [98]. There are many in vitro reports that showed good neuroprotective properties of amifostine against cisplatin and oxaliplatin, and some studies on patients claiming promising effects of amifostine against peripheral neurotoxicity induced by cisplatin, oxaliplatin, and carboplatin [99,100]. Moreover, a network meta-analysis of Fu and coworkers [76] confirmed that amifostine was the most active against both overall and severe platinum drugs-induced neurotoxicities compared to other most used therapies, such as vitamin E, GSH, and Ca/Mg infusion. Metformin, an anti-diabetic drug, showed neuroprotective effects against other chemotherapy-induced neuropathies, and therefore it has recently been tested in vivo for alleviating the oxaliplatin-induced neuropathy in rats. It was able to decrease levels of ROS and markers of oxidative stress and to ameliorate intraepidermal fibers degeneration, gliosis, and sensitivity [77]. The new drug in the treatment of multiple sclerosis - dimethyl fumarate (DMF) was tested because of its neuroprotective properties on cisplatin and oxaliplatin-induced neurodegeneration in the in vitro study [78]. DMF induced up-regulation of the nuclear factor-erythroid-2-related factor 2 (Nrf2)-dependent antioxidant response and prevented the inhibition of neurite outgrowth. The antioxidant and mitoprotective potential of carvedilol, an antihypertensive drug, was tested in vitro on neuronal cells (Neuro-2a) affected by oxaliplatin. Carvedilol is a non-selective beta-adrenergic receptor blocker (β1,β2) of the third generation, and alpha adrenergic receptor blocker (α1) exerting antioxidant potential. It showed significant antioxidant and free radical scavenging effects with the alleviation of functional and sensorimotor deficits, but without in vitro affecting the anti-tumor effects of oxaliplatin [79].

Cisplatin-induced neurotoxicity in rats was treated with oxytocin, a neurohypophyseal nonapeptide synthesized in the hypothalamus [80]. The results showed that oxytocin was able to reduce oxidative stress and inflammation in rats, but also to mitigate the electromyography (EMG) changes in rats treated with cisplatin. Another compound that can reduce the oxidative stress induced by oxaliplatin in rats is phosphatidylcholine [81], but also exhibited potential in the regulation of microglial activation and thus decreasing peripheral neuropathy in rats. In the study of Chiu et al. [34] chemotherapy (cisplatin)-induced cognitive impairment was treated with pifithrin-μ, an inhibitor of mitochondrial p53 accumulation. The use of this small molecule led to a significant improvement in preserving neuronal mitochondrial function. Patients with colorectal cancer treated with oxaliplatin-based chemotherapy were receiving co-treatment with monosialotetrahexosyl- ganglioside, known for its impacts on neuronal plasticity and repair mechanisms, and the release of neurotrophins in the brain [82]. This medication was able to significantly reduce the incidence of neuropathy induced by oxaliplatin, particularly severe neuropathy, with the absence of interfering with oxaliplatin activity.

N-Acetylcysteine (NAC) is a precursor of glutathione which can increase the concentration of glutathione in blood and thus increase the antioxidative defense in the organism [99]. The effects of NAC were studied in cisplatin-treated rats showing reduced levels of oxidative stress parameters in rodents as well as significant anxiolytic properties [83]. NAC was also potent in regulating cognitive performance and improving the histomorphological parameters that were disrupted by cisplatin administration [74]. Some PARP inhibitors may also be used for alleviating the chemotherapy-induced neurotoxicities. Since oxidative stress and mitochondrial dysfunction are closely connected with side effects of platinum chemotherapy-induced neuropathy, the inhibition of poly(ADP-ribose) polymerase (PARP), would be beneficial in terms of sensory and enteric neuroprotection [84]. Thus, several PARP inhibitors, such as GPI 21016 and ABT-888 analogue, showed alleviated sensory and enteric neuropathy against cisplatin and oxaliplatin therapy [84].

Many studies have dealt with the neuroprotective activity of different medications against platinum drugs-induced neuropathy. Several compounds were considered to have a number of favorable properties. For example, an chemoprotectant BNP7787 showed good neuroprotective effects in phase II randomized study [99]. A therapy based on chelation properties can be used in the treatment of metal overexposures, including platinum, supporting their excretion from the body. Generally, based on the Lewis theory of hard and soft acids and bases, Pt^2+^ as a soft acid (soft metal) has a high affinity for chelation with compounds possessing soft bases (soft ligands) [101]. For example, the organosulfur complex diethyldithiocarbamate (DDTC) was thought to be able to act as an antioxidant by chelation of Pt^2+^ ions, increasing renal excretion of platinum thereafter influencing on neurophysiological aspects of patients treated with cisplatin. But in the randomized placebo-controlled multicenter evaluation of DDTC in cisplatin treatment, it was shown that DDTC was not able to alleviate cisplatin-induced toxicities [87]. On the other hand, a chelation complex fodipir (DPDP), a derivative of vitamin B6, acts by lowering oxidative and nitrosative stress in PIPN patients. The probable mechanism of PIPN development is via retention of Pt^2+^ ions in the dorsal root ganglion and where they can bind to proteins, that lead to the development of oxidative and nitrosative stress [102]. In the recent study of Stehr et al. [88] was shown that Pt^2+^ has an affinity for DPDP, and the newly formed complex of metal with DPDP has fast excretion and low toxicity which are positive aspects for use of DPDP as chelation therapy of PIPN. Another chelation complex of manganese and the ligand fodipir, mangafodipir, displayed significant results in the study conducted by Coriat et al. [89] on oxaliplatin-induced neurotoxicity in a mouse model as well as in patients with oxaliplatin neuropathy (grade ≥2). This complex is generally inactive, yet while it is indicated that it has to be subjected to metabolism into N,N’-dipyridoxyl ethylenedi amine-N,N’-diacetic acid (MnPLED) in organism so it can show its cytoprotective effects. The results of the above-mentioned research showed that intravenous administration of mangafodipir along with oxaliplatin led to the alleviation of oxaliplatin-induced peripheral neuropathy in patients with grade 2 neuropathy and thus can be a successful choice for adjuvant therapy. A mixed metal complex calmangafodipir (Ca^2+^/Mn^2+^) was patented in 2015 by Karlsson et al. [90] as a new stable complex, whose toxic effects are reduced compared to mangafodipir, acting as a reducer of oxidative stress and levels of reactive oxygen species. Thus it became the object of different investigations, one of which dealt with its activity on oxaliplatin-induced peripheral neuropathy in a placebo-controlled randomized phase II study [103]. The authors concluded that calmangafodipir was able to prevent the development of oxaliplatin-induced peripheral neuropathy during and after treatment, at a dose of 5 mmol/kg. However, Karlsson & Jynge [104] criticized the implementation and interpretation of the results by Glimelius et al., proposing a revision of methodology and design of the experiment (phase II study) so that it can be accurate and authoritative for the further development of a phase III study. There are still no approved chelation-based treatments of PIPN, but research work is encouraged to find appropriate therapy without affecting the platinum-drug activity and without side-effects [101].

Xaliproden, a 5HT1A agonist that possesses neuroprotective activity, showed a reduction in grade 3–4 neurotoxicity by activation of MAPK pathways in oxaliplatin-induced neuropathy without affecting FOLFOX4 antitumor activity in first-line treatment of patients with metastatic colorectal cancer (MCRC) [91]. Some new perspectives of drug preparation and application are being investigated. Therefore, basalin-coated silver nanoparticles were developed and applied as a potent antioxidant for the amelioration of oxaliplatin-induced peripheral neuropathy in mice [92]. They were able to significantly perform the chelation of aluminum and consequently ameliorate neuropathic pain in mice.

### 5.3. Natural Products and Medicinal Plants

Compounds of the natural origin are in use in traditional and modern medicine regarding their numerous beneficial effects. They can be used in the prevention and/or therapy of various pathological conditions such as cardiovascular diseases, metabolic disorders, carcinogenesis, and neural impairments [105]. The most widely studied phytochemicals are definitely phenolic compounds or polyphenolics from medicinal plants. They exerted a wide range of biological activities, including antioxidant, anti-inflammatory, anti-apoptotic, and anti-cancer effects, which can be the main pillars of their actions as neuroprotectors, particularly towards platinum drugs-induced neurotoxicities (Table 3) [48,106,107,108,109]. One of the well-studied phenolic compounds with neuroprotective properties is curcumin (Figure 2), a yellow pigment isolated from *Curcuma longa* (Zingiberaceae) rhizomes. It showed protective activity in vitro, in terms of neurite outgrowth inhibition in PC12 cells treated with cisplatin, but on the other hand, curcumin had not affected the anticancer activity of cisplatin in HepG2 tumor cells [110]. The in vivo studies of Oz et al. [111] in rats placed curcumin as a compound that significantly alleviated cisplatin-induced learning and memory impairments. It was able to lower the oxidative stress level and improve the cognition and cholinergic functions affected by cisplatin. These results in different models suggested that curcumin may diminish cisplatin-induced neurotoxicity. Apart from its properties against cisplatin, curcumin can be used to prevent the neurotoxicity induced by oxaliplatin. It was able to significantly restore enzymatic and non-enzymatic antioxidant capacity and to alleviate the mitochondrial activity in the brain mitochondria of rats as well as to lower neurotensin plasma concentration, improve motor and behavioral parameters and reduce the concentration of oxaliplatin in the sciatic nerve of male Wistar rats [112,113].

Flavonoids, the most common class of polyphenolic compounds in the plant kingdom, are well-known for their biological potential which mostly lies in the fact that they have exceptional antioxidant properties [140]. Quercetin, a bioactive flavonol, and its glucoside rutin (quercetin-3-*O*-rutinoside) (Figure 2) were tested for their ability to restore increased thermal and mechanical nociceptive response induced by oxaliplatin in mice [114]. Both compounds significantly reduced oxidative stress and prevented oxaliplatin-induced chronic painful peripheral neuropathy. Rutin confirmed its neuroprotective action against cisplatin in an in vivo study conducted by Almutairi and coworkers [115]. The high expressions of PON-1, PON-3, PPAR-δ, and GPX in rat brain tissues caused by cisplatin were restored by rutin application while PON-2 expression levels were increased. It was clear that rutin manifests its activity via the antioxidant pathway. Another flavonoid compound, 6-methoxyflavon, was tested against cisplatin-induced neuropathic allodynia and hypoalgesia in rats [116]. This compound exerted both peripheral and central antinociceptive activities, reducing the chemotherapy-induced peripheral neuropathy, but with the absence of motor side-effects characteristic to gabapentin as a control.

Phenolic acids are known for their significant antioxidant, antitumor, and antimicrobial activity and thanks to that they display many benefits on human health [141,142]. Many members of this group of phenolics showed powerful effects against different neurological disorders acting as neuroprotectors [143]. These compounds also showed significant alleviation of disrupted parameters during the cisplatin treatment, particularly rosmarinic acid [144], ellagic acid [145], protocatechuic acid [146], and caffeic acid phenethyl ester [147]. Therefore, it is not a surprising fact that rosmarinic acid (Figure 2) was able to mitigate mitochondrial dysfunction and spinal glial activation in vitro and in vivo in oxaliplatin-induced peripheral neuropathy [119]. Salicylic acid (Figure 2) showed a reduction in cisplatin neurotoxicity by antioxidant effects in rat primary neuron cell cultures in vitro [117]. Moreover, in cisplatin-induced neurotoxicity in rats, caffeic acid phenethyl ester, a derivative of caffeic acid, was capable to restore to normal activities of brain metabolic enzymes (hexokinase, glucose-6-phosphate dehydrogenase, lactate dehydrogenase, and malate dehydrogenase) showing vital activity against the development of neuropathy [118].

In the study of Li et al. [120] cyanidin, a natural phenolic compound belonging to the group of anthocyanidins present in many fruits and vegetables, especially colored berries, cherries and grapes (Figure 2), was able to suppress oxidative stress in cisplatin-induced neurotoxicity on PC12 cells based on its notable antioxidant potential against ROS overproduction. The main bioactive compound of *Nigela sativa* (black cumin) seed oil is thymoquinone (Figure 2). Due to its antioxidant, anti-inflammatory, anti-neoplastic, and neuroprotective properties, it exhibited protective activity against cisplatin neurotoxicity in cultured DRG neurons [121]. Namely, thymoquinone promoted the neuronal cell viability and neurite outgrowth in a dose-dependent manner. In another in vivo study, thymoquinone reduced oxidative stress downregulated the apoptotic markers (p38 mitogen-activated protein kinase (MAPK), STAT-1, p53, p21, and MMP9) in rats and protected brain tissue against cisplatin action [122]. In the same study [122], geraniol, monoterpenoid alcohol, showed similar effects as thymoquinone during cisplatin-induced neurotoxicity in rats. Chen et al. [123] studied the effects of ginsenoside Rb1, a ginsenoside found in *Panax ginseng* and *Panax japonicus* var. *major* roots, on cisplatin-induced memory impairments in rats. In assays such as novel objects recognition task and Morris water maze task it was shown that ginsenoside Rb1 significantly ameliorated memory impairments caused by cisplatin, as well as reduced the neuronal loss induced by cisplatin in different regions (CA1, CA3, and dentate gyrus) of the hippocampus. Moreover, this compound exhibited the ability to rescue the cholinergic neuron function in rat brain and also lowered oxidative stress and neuroinflammation.

Isothiocyanates, a group of natural bioactive compounds mostly present in cruciferous vegetables, are characterized by their antioxidant properties [148]. That is what enabled them to be used in the alleviation of platinum drug-induced neurotoxicity. Allyl-isothiocyanate alleviated neuropathic pain induced by oxaliplatin in rats and reduced the hypersensitivity to cold non-noxious stimuli by releasing H_2_S [124]. Similarly, glucosinolate glucoraphanin and derived isothiocyanate sulforaphane exerted reducing effects on oxaliplatin induced-neuropathic pain in mice, in a dose-dependent manner by releasing H_2_S and modulating Kv7 channels [125]. The same research group, Di Cesare Mannelli et al. [126] conducted in vivo experiments on oxaliplatin-induced neuropathy using silibinin as an antioxidant compound. Silibinin (Figure 2) is a flavonolignan isolated from *Silybum marianum* that possesses antioxidant and antineoplastic activities. In this study, silibinin reduced oxidative damage of oxaliplatin in rats at a concentration of 100 mg/kg. It also recovered motor coordination and showed a reduction in oxaliplatin-dependent pain induced by mechanical and thermal stimuli. Silibinin is one of the components in silymarin complex mixture which also contains three more flavonolignans (silychristin, silydianin, and taxifolin) and its use is mainly focused on liver disorders treatment [149]. Silymarin in rats, at a concentration of 100 mg/kg, decreased the anxiogenic effect of cisplatin treatment and exhibited significant antioxidant activity in brain tissues by lowering lipid peroxidation and elevating GSH levels and CAT and SOD activities [127].

Many herbal mixtures are in use for relieving chemotherapy-induced neuropathic pain. One that showed significant improvement regarding the negative effects of platinum-based drugs is goshajinkigan, a traditional Japanese Kampo medicine consisted of ten medicinal plants that is used clinically to treat pain in Japan. Ushio et al. [128] showed that goshajinkigan was able to prevent cold hyperalgesia and mechanical allodynia during the oxaliplatin-induced neuropathy in rats. The treatment with goshajinkigan also exerted significant improvements of oxaliplatin neuropathy in non-resectable or recurrent colorectal cancer patients [129]. Moreover, in phase 2, multicenter, randomized, double-blind, placebo-controlled trial of goshajinkigan was shown its benefits towards preventing oxaliplatin-induced neuropathy without affecting the activity of the chemotherapeutic [130]. Wei and coworkers [131] monitored the neuroprotective effects of some mixtures of Traditional Chinese Medicine against oxaliplatin in cancer patients. The highest neuroprotective potential had Huangqi Injection which consisted of an extract of *Astragalus membranaceus radix* and Huangqi Guizhi Wuwu Decoction that contained a mixture of *Astragalus membranaceus radix*, *Cinnamomum cassia*, *Paeonia lactiflora*, *Ziziphus jujuba*, and *Zingiberis recens rhizoma*. The common feature for both mixtures was *Astragali radix* (Huang Qi). The extracts of *Astragalus* roots, aqueous and two hydroalcoholic extracts, were tested in vitro against oxaliplatin-induced neurotoxicity in the neuronal-derived cell line SH-SY5Y and in primary cultures of rat cortical astrocytes [132]. The *Astragali radix* extracts exhibited strong antioxidant activity, ameliorating the lipid peroxidation, proteins, and DNA oxidation. The 50% hydroalcoholic extract was dominant in the prevention of caspase-3 activation and it stimulates astrocyte viability.

Another immensely important medicinal plant in traditional Chinese medicine is Danshen or *Salvia miltiorrhiza*, mainly used in the treatment of cardiovascular diseases and neurasthenic insomnia [150]. Nevertheless, Danshen and its active constituents tanshinones (tanshinone IIA and cryptotanshinone) exhibited promising activity against oxaliplatin-induced neuropathic pain where they reduced chemotherapy-induced nociceptive hypersensitivity in mice and attenuated glioblastoma cells malignancy. Danshen and tanshinones had the long-lasting pain-relieving effects so they could serve as adjuvant therapy of choice in the oxaliplatin treatment. Another plant tested, because of its significant antioxidant potential, in the model of cisplatin-induced neuropathy in mice, was chamomile (*Matricaria chamomilla*). The ethanol extract of chamomile flowers showed anti-inflammatory effects and reduction of pain responses in the formalin test in mice with intensive analgesic effect [134]. *Hypericum perforatum* L. (St. John’s wort) is well-known for its antioxidant, anti-inflammatory, analgesic, and neuroprotective effects [151,152,153]. Its extract showed significant protective activity against oxaliplatin-induced neurotoxicity in vitro and reduced caspase-3 activity in rat astrocytes without the reduction of oxaliplatin cytotoxicity on HT-29 cells [135]. *Satureja hortensis* aerial part extract, applied in rats at three different concentrations (50, 100 and 200 mg/kg) along with cisplatin injection, exhibited strong anxiolytic activity and lowered apoptotic parameters in rat hippocampal tissues [127]. Moreover, it was able to significantly reduce the oxidative stress in brain tissues by alleviating CAT and SOD activities and GSH levels and decreasing the levels of lipid peroxidation indicators.

*Vitis vinifera* L., the common grapevine, is known for many benefits on human health, particularly for the antioxidant potential of its main bioactive compounds proanthocyanidins but also many phenolic acids and flavonoids [106,141]. The *V. vinifera* hydroalcoholic extract in the model of oxaliplatin-induced neurotoxicity showed extensive activity towards the reduction of superoxide anion concentration and lipid peroxidation in rat astrocytes [136]. It also suppressed mechanical and thermal hypersensitivity in rats while a decline of astrocytes activation in the spinal cord was monitored. The grape seed proanthocyanidin extract also showed neuroprotective power against carboplatin in a study reported by Yousef et al. [137]. The main way of acting was through decreasing the oxidative stress in brain tissue and reducing cytokines, p53, neurotransmitters and biochemical parameters. The extract also inhibited brain cell apoptosis and alleviated carboplatin effects on the histological parameters. Green tea (*Camellia sinensis*) can attenuate toxicities linked to treatment with platinum-based drugs (cisplatin, oxaliplatin) [154]. Its extract had notable potential on oxaliplatin-induced peripheral neuropathy in rats where caused alleviation of sensory symptoms after oxaliplatin treatment, such as allodynia, but it was inefficient in the prevention of morphometric or electrophysiological alterations [138]. *Lithospermum erythrorhizon* is a plant used in traditional Chinese medicine for eczema and other skin diseases as well as for wound healing. However, it was shown that *Lithospermi radix* extract can be an excellent neuroprotective agent against oxaliplatin-induced peripheral neuropathy in both in vitro and in vivo models [139].

## 6. Conclusions

In summary, we intentionally presented the variety of previously reported beneficial effects of antioxidant supplementation in the treatment of neurotoxicity, as one of the most frequent dose- and time-limiting factors in chemotherapeutic protocols that include platinum-based compounds. It seems reasonable that future investigations that should analyze the protective role of specific antioxidants more precisely may result in achieving even more subtle coverage of chemotherapeutic protocols with adverse effects preventing strategy.

## Figures and Tables

**Table 1:** Classification of antioxidants.

Endogenous Antioxidants		Exogenous Antioxidants
Enzymatic	Non-Enzymatic	Non-Enzymatic
Catalase (CAT)	Myoglobin	**Vitamins**
Superoxide dismutase (SOD)	Ferritin	Vitamin C (ascorbic acid)
Glutathione peroxidases (GPxs)	Metallothioneins	Vitamin E (tocopherols)
Glutathione reductases (GRs)	Transferrin	Vitamin K
Glutathione-*S*-transferases (GSTs)	Lactoferrin	**Mineral elements**
Phospholipase A2	Albumin	Selenium
	Ceruloplasmin	Zinc
	Glutathione	Manganese
	Coenzyme Q10	Copper
\	Uric acid	**Nutritional**
	Bilirubin	Carotenoids
	Melatonin	Phytonutrients
	Vitamin A (retinol)	**Supplements**
		Lipoic acid
		Polyphenol
		D-Methionine
		Carnosine
		Omega-3 fatty acids
		Acetyl L-carnitine

**Table 2:** Vitamins, dietetic supplements, and medication with neuroprotective activity against neurotoxicity induced by platinum-based chemotherapeutics.

Agent/Compound	Pt-Based Drug	Action	Reference
Retinoic acid (metabolite of vitamin A)	Cisplatin	A mild generalized protective effect in rats’ model.	Tredici et al. [50]
	Cisplatin	Reversed sensorial changes and nerve morphology in rats and reduced axonal degeneration in patients.	Arrieta et al. [51]
Vitamin E	Cisplatin	Protected peripheral nerve and increase antioxidant defense in mice.	Leonetti et al. [52]
	Cisplatin	Ameliorated peripheral neuropathy in patients (a randomized, open label with blind assessment, controlled trial).	Argyriou et al. [53]
	Cisplatin	Reduced neuropathy symptom assessments (phase III clinical trial).	Pace et al. [54]
	Cisplatin, carboplatin, oxaliplatin, or combination	No significant reduction of the incidence of sensory neuropathy (phase III clinical trial).	Kottschad et al. [55]
	Oxaliplatin	No significant decrease in peripheral neuropathy (phase II clinical trial and a prospective randomized, controlled clinical trial).	Afonseca et al. [56] Salehi et al. [57]
WS-CoQ10 (water-soluble formulation of provitamin coenzyme Q10)	Cisplatin	Reduce DNA damage and neuritic toxicity in PC12 cells.	Machado et al. [58]
Thiamine pyrophosphate	Cisplatin	Prevented oxidative stress in rats brain tissue.	Turan et al. [59]
Selenium (sodium selenite)	Cisplatin	Prevented peripheral neurotoxic effect in rats	Erken et al. [49]
Calcium and Magnesium	Oxaliplatin	Prevent neurotoxicity in PC12 cells. Prevent oxaliplatin-induced cold hyperalgesia in rets. Not effective in preventing oxaliplatin-induced sensory neurotoxicity (phase III randomized, placebo-controlled, double-blind study).	Takeshita et al. [60] Sakurai et al. [61] Loprinzi et al. [62]
Alpha lipoic acid (ALA)	Cisplatin	Prevented mitochondrial energetic failure, neuronal apoptosis, and axonal damage in vitro.	Melli et al. [63]
	Cisplatin	Restored electrophysiological parameters of the compound action potential of the rat sciatic nerve.	Tuncer et al. [64]
	Cisplatin or oxaliplatin	No statistically significant differences were found between the ALA and the placebo groups of patients with neuropathic symptoms	Guo et al. [65]
	Cisplatin or oxaliplatin	Reduced neuropathy symptoms in patients (small clinical studies)	Gedlicka et al. [66,67]
L-Carnosine	Oxaliplatin	Reduced peripheral neuropathy in patients with colorectal cancer by reducing inflammation (decreased NF-κB and TNF-α), oxidative stress (reduced MDA and increased Nrf-2) and apoptosis (reduced caspase-3 activity).	Yehia et al. [68]
Ergothioneine	Cisplatin	Restored the cognition in mice possibly through the inhibition of oxidative stress and restoration of AChE activity in neuronal cells.	Song et al. [69]
Ethoxyquin	Cisplatin	Prevented neurotoxicity in vitro in a sensory neuronal cell line and primary rat dorsal root ganglion neurons; ameliorated behavioral, electrophysiological and morphological abnormalities in rats.	Zhu et al. [70]
Melatonin	Cisplatin	Restored electrophysiological parameters of the compound action potential of the rat sciatic nerve.	Tuncer et al. [64]
	Oxaliplatin	Altered the inactivation of apoptotic proteins non-enzymatic, enzymatic antioxidants and complex enzymes of mitochondria.	Waseem et al. [71]
D-Methionine	Cisplatin	Ameliorated the cortical neurons damage in vitro.	Gopal et al. [72]
	Cisplatin	Prevented the decrease of neurogenesis in the hippocampus of the adult rats.	Hinduja et al. [73]
	Cisplatin	Regulated electrophysiological recordings, increased hippocampal neurogenesis in rodents.	Rosić et al. [74]
L-Methionine	Cisplatin	Regulated electrophysiological recordings.	Gopal et al. [72]
Taurine	Cisplatin	Improved behavioral performance and brain antioxidant status of rats	Owoeye et al. [75]
Amifostine	Cisplatin Oxaliplatin	The highest efficacy for both overall and severe neurotoxicities compared to vitamin E, GSH, and Ca/Mg infusion.	Fu et al. [76]
Metformin	Oxaliplatin	Prevented degeneration of intraepidermal fibers, gliosis, and the altered sensitivity in rats.	Martinez et al. [77]
Dimethyl fumarate	Cisplatin Oxaliplatin	Attenuated inhibition of neurite outgrowth, increased Nrf2 DNA binding activity in vitro.	Kawashiri et al. [78]
Carvedilol	Oxaliplatin	Prevented functional deficits in peripheral nerve mitochondria of rats. Reduction in levels of intracellular ROS and mitochondrial superoxide in N2a cells.	Areti et al. [79]
Oxytocin	Cisplatin	Mitigated the EMG alterations, reduced oxidative stress and inflammation, and enhanced antioxidative potential.	Akman et al. [80]
Phosphatidylcholine	Oxaliplatin	Reduced oxidative stress parameters, ameliorated microglial activation and thus reduced peripheral neuropathy in rats.	Kim et al. [81]
Pifithrin-μ	Cisplatin	Inhibited mitochondrial p53 accumulation in vivo.	Chiu et al. [34]
Monosialotetrahexosylganglioside	Oxaliplatin	A significantly lowered incidence of grade 1–3 acute neurotoxicity in patients with colorectal cancer.	Chen et al. [82]
N-acetylcysteine (NAC)	Cisplatin	Significant anxiolytic effect regarding decreased oxidative stress parameters in rats.	Vuković et al. [83]
	Cisplatin	Alleviated cognitive performance, regulated histomorphological parameters, lowered oxidative stress.	Rosić et al. [74]
PARP inhibitor (GPI 21016)	Oxaliplatin	Alleviated sensory and enteric neuropathy via PARP inhibition.	McQuade et al. [84]
PARP inhibitor (ABT-888 analogue)	Cisplatin Oxaliplatin	Alleviated sensory and enteric neuropathy via PARP inhibition.	McQuade et al. [84]
BNP7787	Cisplatin	Improvements in phase II randomized study.	Parker et al. [85] and Hausherr et al. [86]
Diethyldithiocarbamate (DDCT)	Cisplatin	Changed severity and neurophysiological assessment, while lowering cisplatin concentration in patients.	Gandara et al. [87]
Fodipir (DPDP)	Oxaliplatin	Showed high affinity for Pt^2+^.	Stehr et al. [88]
Mangafodipir	Oxaliplatin	Neuropathy improved or stabilized after 4 cycles and after 8 cycles neurotoxicity was downgraded to grade ≥2, in a phase-II study.	Coriat et al. [89]
Calmangafodipir	Oxaliplatin	Reduced ROS accumulation.	Karlsson et al. [90]
Xaliproden	Oxaliplatin	Reducing the risk of grade 3–4 oxaliplatin-induced PSN without impacting FOLFOX4 antitumor activity.	Cassidy et al. [91]
Basalin coated silver nanoparticles	Oxaliplatin	Alleviated neuropathic pain in mice by the chelation of aluminum.	Gao et al. [92]

**Table 3:** Compounds from natural sources, natural products, and medicinal plants with promising protective activity against platinum-drugs induced neuropathy.

Agent/Compound	Pt-Based Drug	Action	Reference
Curcumin	Cisplatin	Reduced neurotoxicity in NGF-differentiated PC12 cells.	Mendonça et al. [110]
	Oxaliplatin	Significant activity in the brain mitochondria and Sciatic nerve of male Wistar rats.	Waseem & Parvez [112] Al Moundhri et al. [113]
	Cisplatin	Improved cognition, oxidative stress, and cholinergic functions in rats.	Oz et al. [111]
Quercetin	Oxaliplatin	Prevented thermal and mechanical nociceptive response and decreased oxidative stress in mice.	Azevedo et al. [114]
Rutin	Oxaliplatin	Prevented thermal and mechanical nociceptive response and decreased oxidative stress in mice.	Azevedo et al. [114]
	Cisplatin	Restored levels of PON-1, PON-3, PPAR-δ, and GPX and significantly increased PON-2 expression.	Almutairi et al. [115]
6-Methoxyflavon	Cisplatin	Good peripheral and central antinociceptive activities against a tonic and phasic nociceptive stimuli.	Shahid et al. [116]
Salicylic acid	Cisplatin	Decreased neurotoxicity by lowered oxidative stress in rat primary neuron cell cultures *in vitro*.	Cetin et al. [117]
Caffeic acid phenethyl ester	Cisplatin	Alleviated activities of the glucose metabolizing enzymes of brain tissue in rats.	Özyurt et al. [118]
Rosmarinic acid	Oxaliplatin	Mitigated mitochondrial dysfunction and spinal glial activation in vitro and in vivo.	Areti et al. [119]
Cyanidin	Cisplatin	Inhibited reactive oxygen species (ROS)-induced DNA damage in cisplatin-treated PC12 cells.	Li et al. [120]
Thymoquinone	Cisplatin	Increased the ability to extend neurites and neuronal cell in primary DRG neurons in vitro.	Üstün et al. [121]
	Cisplatin	Reduced neurotoxicity by downregulating the p38 MAPK/STAT-1 pathway and oxidative stress in rats.	Kandeil et al. [122]
Geraniol	Cisplatin	Reduced neurotoxicity by downregulating the p38 MAPK/STAT-1 pathway and oxidative stress in rats.	Kandeil et al. [122]
Ginsenoside Rb1	Cisplatin	Ameliorated the memory impairments and the neuronal loss, saved the cholinergic neuron function, inhibited the oxidative stress and neuroinflammation in rat brain.	Chen et al. [123]
Allyl-isothiocyanate	Oxaliplatin	Lowered the hypersensitivity to cold non-noxious stimuli, reduced neuropathic pain by releasing H_2_S.	Di Cesare Mannelli et al. [124]
Glucoraphanin and sulforaphane	Oxaliplatin	Reduce neuropathic pain by releasing H_2_S and modulated Kv7 channels in mice.	Lucarini et al. [125]
Silibinin	Oxaliplatin	Reduced pain induced by mechanical and thermal stimuli, alleviated antioxidant defense in rats.	Di Cesare Mannelli et al. [126]
Silymarin	Cisplatin	Induced significant anxiolytic effects with reduced oxidative stress and apoptotic parameters in rat brains.	Kumburovic et al. [127]
Goshajinkigan	Oxaliplatin	Prevented cold hyperalgesia and mechanical allodynia after the development of neuropathy in rats.	Ushio et al. [128]
	Oxaliplatin	Prevented neuropathy in unresectable or recurrent colorectal cancer patients.	Nishioka et al. [129]
	Oxaliplatin	Acceptable safety margin and a promising effect in delaying the onset of grade 2 or greater in patients, a phase II study.	Kono et al. [130]
TCM: Huangqi Injection (an extract of *Astragalus membranaceus radix*) and Huangqi Guizhi Wuwu Decoction (*Astragalus membranaceus radix, Cinnamomum cassia, Paeonia lactiflora, Ziziphus jujuba*, and *Zingiberis recens rhizoma*)	Oxaliplatin	Prevented peripheral neurotoxicity in cancer patients.	Wei et al. [131]
*Astragali radix* (Huang Qi) extracts	Oxaliplatin	Protected against lipid peroxidation, oxidation of proteins, and DNA oxidation. Prevented the caspase-3 activation and stimulates astrocyte viability *in vitro*.	Di Cesare Mannelli et al. [132]
Danshen (*Salvia miltiorrhiza*) and tanshinones	Oxaliplatin	Attenuated chemotherapy-induced nociceptive hypersensitivity in mice.	Di Cesare Mannelli et al. [133]
*Matricaria chamomilla* extract	Cisplatin	Decreased inflammation and pain responses in the first and, particularly, second phase in formalin test in mice.	Abad et al. [134]
*Hypericum perforatum* extract	Oxaliplatin	Reduced caspase-3 activity in rat astrocytes *in vitro*.	Cinci et al. [135]
*Satureja hortensis* extract	Cisplatin	Induced significant anxiolytic effects with reduced oxidative stress and apoptotic parameters in rat brains.	Kumburovic et al. [127]
*Vitis vinifera* extract	Oxaliplatin	Reduced levels of superoxide anion and lipid peroxidation in rat astrocytes, prevented mechanical and thermal hypersensitivity in rats, decreased the activation of astrocytes in the spinal cord.	Micheli et al. [136]
Grape seed proanthocyanidin extract	Carboplatin	Acted on brain cytokines, P53, neurotransmitters, reduced oxidative stress, lowered histological changes.	Yousef et al. [137]
*Camellia sinensis* extracts	Oxaliplatin	Alleviated sensory symptoms in rats after therapy.	Lee et al. [138]
*Lithospermi radix* extract	Oxaliplatin	Showed anti-inflammatory activity in the spinal cord and protective effects in the peripheral nerve system.	Cho et al. [139]
